# Meanings of existential uncertainty and certainty for people diagnosed with cancer and receiving palliative treatment: a life-world phenomenological study

**DOI:** 10.1186/1472-684X-13-28

**Published:** 2014-06-11

**Authors:** Magdalena Karlsson, Febe Friberg, Catarina Wallengren, Joakim Öhlén

**Affiliations:** 1 Institute of Health and Care Sciences, Sahlgrenska Academy at the University of Gothenburg, Sweden, Box 457, Gothenburg SE- 405 30, Sweden; 2 Sahlgrenska University Hospital, Per Dubbsgatan, Gothenburg SE- 413 45, Sweden; 3 University of Gothenburg Centre for Person-Centred Care, Box 457, Gothenburg SE- 405 30, Sweden; 4 Department of Health Studies, Faculty of Social Sciences, University of Stavanger, Stavanger 4036, Norway; 5 Palliative Research Centre, Ersta Sköndal University College and Ersta Hospital, Stockholm, Sweden

**Keywords:** Existential uncertainty and existential certainty, Life-world phenomenology, Nursing, Palliative care, Personal growth, Qualitative research, Spirituality

## Abstract

**Background:**

Many people around the world are getting cancer and living longer with the disease. Thanks to improved treatment options in healthcare, patients diagnosed with advanced gastrointestinal cancer can increasingly live for longer. Living with cancer creates existential uncertainty, but what does this situation mean for the individual? The purpose of the study is to interpret meanings of existential uncertainty and certainty for people diagnosed with advanced gastrointestinal cancer and receiving palliative treatment.

**Methods:**

This study is part of a larger project in which 7 men and 7 women aged between 49 and 79 participated in a study of information and communication for people with advanced gastrointestinal cancer. A total of 66 interviews were conducted with participants who were followed up over time. The narrative interviews were transcribed verbatim and the texts were analysed in three steps: naive reading, structural analysis and interpreted whole by utilizing a phenomenological life-world approach.

**Results:**

This study has identified different spheres in which people diagnosed with advanced gastrointestinal cancer vacillate between existential uncertainty and certainty: bodily changes, everyday situations, companionship with others, healthcare situations and the natural environment. Existing in the move between existential uncertainty and certainty appears to change people’s lives in a decisive manner. The interview transcripts reveal aspects that both create existential certainty and counteract uncertainty. They also reveal that participants appear to start reflecting on how the new and uncertain aspects of their lives will manifest themselves –a new experience that lays the foundation for development of knowledge, personal learning and growth.

**Conclusions:**

People diagnosed with advanced gastrointestinal cancer and receiving palliative care expressed thoughts about personal learning initiated by the struggle of living with an uncertain future despite their efforts to live in the present. Their personal learning was experienced through a changed life for themselves and having to confront their own pending death and develop self-insight regarding finality of life. Healthcare professionals can try to support people receiving palliative treatment for cancer by diversifying avenues for their personal growth, thus helping them manage their existential uncertainty and gravitate towards greater existential certainty.

## Background

Suffering in relation to living with cancer often creates uncertainty. Uncertainty is frequently described as a sense of having a low degree of confidence in and poor control over various aspects of life, while certainty conversely relates to a high degree of the same [1]. Research reveals that people diagnosed with suspected cancer feel uncertain about whether or not they have cancer [2], the effectiveness of the treatment [3], the results of tests and examinations [4], their scope to achieve personal goals [5], getting through everyday activities [6] and about whether or not they will actually survive [7].

Since suffering is about experiencing a threat to one’s existence [8,9], uncertainty needs to be related to the existential dimension of life. According to Heidegger [10], to exist as a human means “being-in-the-world”; an integration of the life and the world. Merleau-Ponty [11] further developed this ambivalent and complex relationship as an embodied existence where the lived body is regarded as the starting-point for our experiences and actions [12]. Hence, to live in health and illness means to exist with body (breast, digestion), emotions (fear, happiness), thoughts (understanding, beliefs), social connections (family, friends) and more [13]. In particular when a person becomes ill with cancer, the bodily experience involves becoming aware of one’s own mortality [14] and this awareness means to live in existential uncertainties due to the mortal threat of the body [15]. It follows that the existential uncertainty of individuals in this situation is about the uncertainty of the prospect of the disease trajectory, about being able to survive or not and how life will be before death.

People diagnosed with gastrointestinal cancer report existential uncertainty as early on as their diagnosis [16,17]. Kleem et al. [18] and Khatri et al. [19] demonstrate that this uncertainty can remain present for the rest of their lives, from diagnosis to treatment [20] and in the terminal stage. The fact that existential uncertainty is common is supported by studies conducted on younger people with cancer [5] and for different cancer diagnoses [21-24]. Since cancer is highly prevalent, many people may be living with such existential uncertainty. In 2008, close to 13 million people were affected by cancer, in an equal distribution between men and women, and primary sites in the gastrointestinal organs are among the most common form of cancer in the world [25]. Improved treatment methods mean a larger proportion of people diagnosed with gastrointestinal cancer now survive and greater numbers are living for longer with the disease.

Although existential uncertainty prevails in cancer patients, there are studies which show that they can also experience certainty and even existential certainty in the sense of having a fresh take on life, getting to know themselves better and appreciating their families in a different way [5]. Receiving bad news is well known to be challenging information [26,27], but so is making sense of receiving palliative treatments [28]. Further, in relation to patients’ quest for knowledge in order to deal with advanced cancer, existential uncertainty in connection with life-threatening disease transforms into existential certainty [29]. This state is not static, however, as it is affected by the person’s circumstances and state of disease. Reluctance from physicians to disclose prognostic information [30] would indicate one source of uncertainty. Earlier research clearly shows that while the information needs of patients are marked, they are also complex in relation to fluctuations in the progression of the disease. Further research is needed regarding what the vacillation between uncertainty and certainty, as illustrated by these studies, means to the patient [28,29]. The purpose of this study is to interpret meanings of existential uncertainty and certainty for people diagnosed with advanced gastrointestinal cancer and receiving palliative treatment.

## Methods

### Study design

The present study is a secondary aim of a larger project about information provision among people receiving palliative cancer care [28,29,31] where the fieldwork was informed by life-world phenomenology [12,32]. The analysis was performed according to hermeneutic interpretive principles out of Ricoeur’s theory of interpretation in three inter-related steps: naïve reading, structural analysis and interpreted whole [33-35]. In this way, the theoretical bases considered continuity between description and interpretation [12].

### Participants and setting

People were asked to participate from an oncological outpatient clinic that specialises in palliative care for gastrointestinal cancer. For inclusion in the study, participants had to be diagnosed with advanced gastrointestinal cancer. According to the principles of qualitative method, variation was sought across gender, age, occupation, family constellation and socio-economic factors. Individuals whose primary nurses considered them to be too weak to participate (lacking strength to share and relate their experiences) were excluded.

In total, fourteen men and women aged between 49 and 79 years old were included. These patients had had cancer (primary tumour sites were liver, colon and unknown) for periods of from two months up to two decades. All participants were diagnosed with various metastases. Four had permanent colostomies. Participants were included from one month and up to three years after receiving their diagnosis of advanced cancer and initiation of palliative treatments (mainly cytotoxic drugs every second week and in a few cases, subsidiary radiation therapy and surgery) with active symptom management. All the participants were born in Sweden and most of them lived in urban areas. All of them had children but only three had children at home, one had deceased children, and three were widowed with adult children. The participants had a range of occupational backgrounds, and differed in their employment status (8 were retired, 3 on sick leave and 3 worked part time and part-time on sick leave) and the majority had a history of cancer in the family (for more details, see [28]).

### Data collection

Data collection comprised 66 narrative interviews conducted over a period of two-and-a-half years (with supplementary observations selected to facilitate monitoring of participants over time, the aim of which is reported elsewhere [28]). Narrative interviews were chosen in order to open up topics and receive the participants’ stories of experiencing existential uncertainty and certainty. Of the 66 interviews (conducted by FF and JÖ), 54 were conducted at the outpatient clinic, while the remaining 12 were conducted at home, with all but three participants having repeat interviews, thus contributing to richer descriptions (longitudinal analyses was not performed in the analysis reported here).

The narrative interviews varied in length from 30–120 minutes and most were audio recorded. In some cases, notes were taken. The following topic areas were covered in the interviews: 1. What information the participants needed and wanted to know, 2. How they sought such information, 3. What they asked fellow patients and clinical staff respectively, 4. What they reflected on at home and 5. What their preferences were in relation to information and knowledge (for more details, see [28]).

### Data analysis

The audio recorded narrative interviews were transcribed verbatim and the transcripts were analysed in three stages: naive reading, structural analysis and interpreted whole (in line with Ricoeur’s theory of interpretation [33,35]. The initial naive reading served to generate questions for testing in the further analysis. First, the narratives were read all the way through a number of times. This reading aimed to be as unbiased as possible and focused on the descriptions of the narrators and their reflections, which were used to guide the subsequent structural analyses. The participants presented narratives that revealed uncertainty as well as certainty regarding their changed and still-changing life situations following short- and long-term palliative treatment. The data was subsequently processed by means of NVivo in order to discern meanings and actions in narrative segments dealing with existential certainty and uncertainty. The initial reading of these segments suggested further analysis of meanings guided by the following two analytical questions:

What creates existential certainty and uncertainty?

What does it mean to be in the move between existential certainty and uncertainty?

The following analysis aimed to systematically examine the script in order to identify and clarify potential meaning structures. Each narrative segment was analysed on the basis of the two analytical questions. Firstly, meanings were described for each narrative segment that corresponded to one of the analytical questions. The identified meanings were then analysed on the basis of similarities, differences and how these meanings could be related to one another. This analysis led to the disclosure of two meaning structures.

The interpreted whole means creating a deeper understanding of the meanings that have been extracted and clarified during the structural analysis. The discovered meanings were related to the naive readings, the literature, the research questions and the researchers’ own understanding in order to interpret meanings of the studied phenomenon: meanings of existential uncertainty and certainty for people diagnosed with advanced gastrointestinal cancer and receiving palliative treatment. The interpretation was enhanced by the use of Dewey’s theory of human nature and human conduct [36].

### Ethical considerations

Ethical approval was granted by the Ethical Review Board in Gothenburg (Ref. Ö437-02, S551-03). Participants were asked to consent to repeated interviews. Each participant had an initial meeting with one of the researchers at the outpatient clinic. An unstructured style approach between researcher and participant was adopted in order to more successfully capture the participants’ experiences during the interviews.

## Results

For the participants, existential uncertainty and certainty appeared to change life in a decisive way. Our study interviews contain descriptions of aspects which both create existential certainty and counteract uncertainty. The participants said that they started to reflect on how new and uncertain aspects of life would unfold. Below is a presentation of these spheres of existential uncertainty and certainty, followed by the meanings related to moving between them and finally, an interpreted whole.

### Spheres of existential uncertainty and certainty

#### Bodily changes

Participants experienced existential uncertainty when they could no longer recognise their own bodies, before they had learnt to recognise their own bodies’ limitations, or when they had insufficient knowledge about how their bodies reacted to disease.

“No, I can’t cross that line. Now I feel a bit like, yeah, watch it, that cancer hasn’t been busted, it’s in your body, uh-oh you’re overdoing it again, so you’ll get the cancer back – that’s what it feels like.”

Nevertheless, they could feel existential certainty if they got the opportunity to learn more about their disease and how their bodies reacted and functioned. The more the participants got to know their bodies, the more they learned to recognise their own limitations. This knowledge contributed to their development of new body awareness.

### Everyday situations

The participants’ basic sense of security was grounded in everyday life. It was only when this was no longer obvious or functioning that they realised its worth and could appreciate it. Having everyday routines gives a sense of assurance and security. The participants spoke of these routines as providing meaningful occupation during the day and highlighted them as important.

“In the grand scheme of things, aren’t routines just about getting a little movement each day so you don’t get rooted to the spot.”

Even the opportunity to experience a change of environment in everyday life brought relief to the participants from their existential uncertainty. This can be understood as a kind of passage through time and space, in which intrusive thoughts regarding uncertainty ebb away and everyday life can be understood in a different light. Consequently, a functioning ordinary day that included a change of environment in the form of a trip or visiting someone was associated with assurance. This could then create the conditions for existential uncertainty to transform into increased existential certainty.

“It took more strength this time than it did last time; I’m kind of used to it now. I think like a person living with war – there’s a day-to-day in that too, that ordinary life is day-to-day – it’s a part of the whole thing.”

### Companionship with others

The participants maintained that relief from existential uncertainty can be found in companionship with others. Feeling you are of value to other people was described as meaningful. The participants described how important it was to have friends who shared their difficulties and gave support, as well as affirmed their continued value to others. Further, supportive friends and family members provided a sense of context and greater certainty in belonging to a community. Conversely, existential uncertainty was perceived as greater when participants lacked a place in such a community.

“And so relationships, people, friends are what’s important. To seize the opportunity to meet them… it becomes more and more meaningful…”

### Healthcare situations

The participants related how important it was to receive treatment for their disease. They revealed that they sometimes felt vulnerable in the presence of healthcare staff, especially in situations where they felt exposed. The participants perceived imbalance between themselves and the healthcare staff and felt it was important to retain self-determination and integrity. Existential uncertainty existed in healthcare encounters characterised by obvious power imbalances.

“It’s terribly scary to be subjected to power; it does nothing to promote health, you know.”

When the participants perceived themselves to be in a vulnerable situation, a sense of existential uncertainty arose. Integrity-breaching behaviour on the part of healthcare professionals could also contribute to existential uncertainty. The participants emphasised the importance of knowing the truth and receiving straight facts about their disease, gaining the feeling that *“I think I’ve been told the truth.”* This would result in a satisfactory healthcare meeting that strengthened their existential certainty.

The healthcare professionals’ demeanour was described as an important factor in alleviating existential uncertainty. Body language in particular was highlighted as significant to the participants’ interpretation of their situation, and this increased insight meant they were better able to express what kind of information they desired. Existential certainty was promoted by meetings with healthcare professionals when the participants felt that they were listened to, when the healthcare professionals took their time, answered their questions and treated them as human beings, gave them affirmation in their predicament and generated a positive health visit experience.

### The natural environment

Doing various activities in the natural environment were said to be associated with greater awareness, strength and inner calm. The participants described different kinds of experiences with nature and related that in close proximity to nature they could find existential certainty in their existential uncertainty. For example, some of the participants described how they enjoyed walking quickly *or* slowly in the natural environment, and being close to the sea and perhaps going for a swim. Through the power of each person’s different senses, nature awakened vivid memories within them.

### Meanings related to moving between existential uncertainty and certainty

#### Struggling with an uncertain future: living in the present

The participants stated that the present could be experienced as more certain, as opposed to the future, which appeared more uncertain as a result of living with advanced cancer and receiving palliative treatment. In this dichotomy, they spoke of *now* being the time for them to do something meaningful. Postponing an activity (telephone calls, trips, family get-togethers etc.) may mean that it would not actually happen. For the participants, this implied that plans for the future could be jeopardised because of their life-threatening and unpredictable disease.

“You know nothing about it, no future, neither of us do. So we can’t speculate about it – let’s take one thing at a time, and we have to do it now…”

Being personally involved in planning important things for the future was described by the participants as providing a greater sense of certainty in their existential uncertainty. It was also meaningful to have a goal to look forward to, having something ahead of them that was perceived as meaningful.

“Now I just have one goal, and I’ve had it quite a long time now. It’s my granddaughter’s college graduation.”

Participants were ever hopeful that the disease and symptoms would release its grip now or in a few years’ time. They entertained hope of being cured, making a full recovery or getting to live a few more years so that new treatment methods would have time to develop, and consciously tried to live as well as possible during the disease trajectory. Being able to trust people in their surroundings meant participants experienced greater existential certainty in their uncertainty. Thinking about their own future was considered a demanding task, as such thoughts encompassed great existential uncertainty, in their opinion.

“You always hope for the best for as long as possible”

### Confronting one’s own impending death: developing insight about finite lifespan

Living with advanced cancer and receiving palliative treatments encompasses the knowledge that life is finite. Participants spoke about not knowing when they would die, but knowing that they would sooner or later, and talked about coming to terms with the fact that everyone will die.

“Other people know that they will die; the fact that we will die is the only thing we know, it’s just that I know it in a different way. I suppose it’s not certain I’ll die before them, there’s no guarantee at all – I could live much, much longer.”

Existential uncertainty is contained in a fear that can be related to the unpredictable reality. They expressed knowledge of the fact that had they fallen ill maybe five to six years earlier, they would not have been alive today, as the treatment they were receiving had only been available in the last few years. Participants also expressed a sense of feeling privileged that there are more treatment options today.

### Changing one’s view on life and self: experiencing personal growth

Living with a life-threatening cancer was highlighted by some participants as having resulted in a completely new attitude to life and self-insight, and they expressed having experienced personal growth in a positive sense during their period of illness. Existential uncertainty had raised the question of what was important in their individual lives and promoted the insight that there is no point in wasting time on boring things – it was more important to choose things that were interesting and fun.

“I don’t do any boring things… I do what I think is fun.”

Some participants talked about having learnt new life skills that they could share, and also of an increased understanding of how they personally reacted in different crisis situations.

“It’s probably because I’ve gone through so much in my life, so many crises, that you learn to handle it in a way, I think.”

One woman described how she had grown as a person and gained a completely new awareness in life. The gravity of the situation gave her opportunity to reflect on what it meant to live with a cancerous disease in a palliative phase.

“Yes, you take charge of it. I think something good can come from this, that I’ve grown as a person too. I have a completely different awareness now.”

The participants further related how they had gained a wider perspective on life, as well as time for reflection, which they felt they did not have time for while they were working prior to the onset of advanced cancer. They spoke of an insight into and knowledge of the fact that they previously had not given themselves time for important things. This personal maturity and insight was thus connected to an opportunity to gain inner strength and self-respect.

"…that I’ve tried to turn this period of illness around… I can benefit from when I hopefully get…and hopefully have an inner strength with increased self-respect.”

Personal growth also meant finding different ways of being able to handle existential uncertainty and feeling as well as possible in the changed life situation as a result of advanced cancer and palliative treatment.

“*…I’ve worked my way out of certain crises. You throw yourself into work in that regard too. So you don’t have to think.”*

Positive thinking did not mean surviving the cancer but filling the remaining time with many positive values and experiences. A positive attitude to life provided a firm foundation for existential certainty, and uncertainty gained its polar opposite. In this way, reflection on the polarity and oscillation between existential certainty and uncertainty promoted personal growth.

### Interpreted whole

It seems like living with an uncertain future while facing one’s own imminent death gives opportunities for personal growth. This was manifest in situations where patients could, as a result of their disease, familiarise themselves with their changed bodies, the ordinariness of everyday situations, companionship with others, healthcare situations, and the natural environment. Moving between existential uncertainty and certainty encompasses meanings of living an uncertain future, confronting one’s own pending death and experiencing personal growth.

Existential uncertainty and certainty in connection with palliative cancer treatment means that the person’s former life, with all its different habits and experiences, changes in a critical way, allowing a process of personal development. When a change occurs, the person starts to reflect on how the new and uncertain aspects of life will unfold. Existential uncertainty becomes clear when life changes direction from the path the person was previously following. What we mean by personal development in this context can be interpreted with the help of according Dewey’s theory regarding human nature and conduct [36] a human is seen as an experiential person who lives and acts and thereby develops in the interplay with other humans. Both learning and new habits are created in order to cope with the challenges of life. Times of great life change give humans the opportunity to engage in reflective problem-solving in order to manage their own changed life situations. The participants in this study highlighted various difficulties and losses that their disease entailed but also that they could observe and learn from. According to Dewey, critical events in life give people new experiences that in themselves lay the foundation for development of knowledge. In this study, we have interpreted this as personal learning and growth. Awareness of the movement between existential uncertainty and certainty and what this movement means promotes personal growth. With reference to Dewey, we could refer to it as a habit that has changed in order to cope with existence. The personal learning and growth that develops does so against a background of living an uncertain future and confronting one’s own pending death. It is learning through a personal lesson about your existence, as well as a lesson about yourself as an experiential person. Against this background, personal maturity and growth can be possible.

## Discussion

### The method

In palliative care, the patients’ narratives are of great importance in understanding how they find increased understanding of and meaning in their situation. The narrative in itself has a healing effect on the patient [37] and assists in creating meaning, identity and a context for their situation [29].

The participants did not use the specific terms existential uncertainty and certainty. However, the interpretation of the data revealed various layers to the phenomenon of existential uncertainty and certainty. Many underlying meanings were contained within the data, although these could be framed and analysed with the interpretive approach in which Ricoeur’s interpretive theory could be adapted to the material [12]. The results can and should be seen as one possible interpretation. Throughout the project, the authors maintained a dialogue amongst themselves to facilitate a critical examination that supports the analysis of the results.

Since weak patients lacking energy and strength to participate in narrative interviews were excluded, the results may not be transferable to dying people or those lacking the strength to share their experiences in a narrative format. Only one narrative interview was performed during the patient’s last week of life.

## Results

The results reveal that when the participants talked about having successful day-to-day routines, they experienced a basic sense of assurance which formed a prerequisite for moving from existential uncertainty to existential certainty. The participants had a desire to live as normally as possible and still feel needed and have something meaningful to occupy themselves with. Everyday routines provided them with a sense of assurance and security. Things that were considered normal in everyday life were habits and routines that the patients could maintain, although these habits may have had to alter somewhat for them to be feasible and comfortable in their situation. It was important that they could share in day-to-day activities as long as possible. The participants tried to establish different areas in which they could experience assurance in their new life situation and thus perceive greater existential certainty.

Research has shown that everyday routines are important [38,39]. The results demonstrate that everyday rituals provide stability in the day-to-day and in life, enhance a person’s quality of life and can give a sense of assurance to patients with advanced cancer [38]. It is when, due to disease, these daily routines are no longer a given fact that a person’s self-image and identity can be threatened [39]. It is when the assurance of the day-to-day is gone and it becomes tinged with uncertainty that unfamiliar feelings and thoughts become part of their normal day and they notice their own existential uncertainty [24]. A major task for healthcare professionals is to make things easier for the person in palliative care and for their relatives, so that they, on the basis of their own wishes, can live out their remaining time together as best they can [40-42]. Further, it is important that healthcare professionals are conscious that patients can move between experiences of existential uncertainty and certainty, as well as having needs or distress of other kinds related to the existential or spiritual dimension of life [43], including unbearable suffering [44].

For our participants it was important to continue to feel valued and part of a community. The results show that existential uncertainty was alleviated when they focused on the present in socialising with family and friends. The participants found it difficult to continue to participate in their work community once they were in the palliative phase of their cancer. Not having functioning relationships through employment made it more difficult for the participants to manage existential uncertainty. If there is no natural community and social context surrounding patients, it is important the healthcare professionals highlight this by conversing with them about opportunities for new social contexts. Sand [24] and Öhlén et al. [45] demonstrate similar results; individuals especially start thinking more about existential questions and concerns with the onset of serious illness. These vast and complex questions have no obvious answers but like most people in a vulnerable situation, the individuals in this study had a need to share their questions and experiences with others. Different forms of companionship are important and relief from existential loneliness can occur through a shared community with other people. Carlander [39] and Friberg and Öhlen [29] likewise show that patients suffering from life-threatening disease find it meaningful to be part of a community and a context; relations with family and friends become important in ensuring the well-being of the patient.

The participants in this study described the natural environment as a place where they could find greater existential certainty in their uncertainty. Similar results have been shown by Sand [24] where spending time with animals and nature is described as important for patients with advanced disease to feel as well as possible. Despite serious illness, it is important for the patient to continue to experience different things, for example, through nature, art and new environments [40,46]. Could it be that ‘nature prescriptions’, beautiful outdoor environments, pictures of nature or virtual nature scenes in healthcare settings are a step in an appropriate direction?

The participants described how meaningful it was to live in the present when they perceived themselves as having an uncertain future. In this study, they highlighted the fact that they had matured on a personal level and consequently developed a new attitude to life and self-insight. They also highlighted different ways of reaching greater existential certainty in their uncertainty and thus becoming more aware of the movement between existential uncertainty and existential certainty. For the participants in this study, acquiring life wisdom was about personal learning and growth. Here we interpret this as learning about one’s existence while learning about oneself as an experiential person. Thus, the present study contributes to and deepens the previous case study in the project about a person’s changing knowledge-seeking foci and related learning outcomes during the illness trajectory at the end-of-life [29]. It also deepens understanding of the results of another study from the project [28], specifically concerning patients’ making sense of palliative treatment as a process of human learning at end-of-life. This was characterized by searching for knowledge and understanding combined with approaches taken as dialectic of living in wait and living in the present [28]. The contribution from the present study specifically highlights spheres of significance for the knowledge-seeking and sense-making processes and thereby possibilities for personal growth.

When life is uncertain, for example, in connection with severe illness, certain things are perceived to be more meaningful than others. Research shows that patients try to grasp, understand and realise the incomprehensible in the approach to end of life and learning in this unique situation encompasses bodily, existential, social and cultural dimensions [29]. People have different ways of managing their situations and should be given support through sensitivity and respect, rather than by being governed and dictated to by others [24]. It is important that the care of seriously ill patients stems from their needs, feelings, thoughts and wishes in the current situation, as well as their wishes regarding how to live out their last days [39]. For a person living with a cancerous disease and undergoing palliative treatment it becomes important to ask how healthcare professionals address the individual patient’s needs in trying to understand what is happening to them and their bodies. Significant questions to highlight are, for example: Are the patients themselves aware of or concerned about the personal growth they are going through? Are they aware of the life skills that they have and how do they become aware of these? Who can guide the individual to this end? Can all patients cope with awareness in this regard?

Current research nevertheless points to a gap between knowledge that pertains to existential questions for a patient’s quality of life in palliative care and that which pertains to daily nursing care [47]. Palliative care policy and guideline documents tend to cover existential issues to a much lesser degree than symptom distress [48]. However, when existential questions are not addressed by healthcare professionals, or addressed in an inappropriate manner, patients may perceive a threat to their identities. More research is required to bridge this gap between existing knowledge and clinical practice. By giving affirmation, listening to patients’ stories, being there and trying to help patients move forward, healthcare professionals can support them in gaining greater existential certainty in their uncertain life predicament. Healthcare can be further improved by, for example, training in this area, reflective discussions and supervision. This contributes to healthcare professionals increasing their awareness not only of the patients’ bodily and existential needs but also of their own attitudes and view of death.

## Conclusions

Living with advanced gastrointestinal cancer and receiving palliative treatment means living with existential uncertainty. This study has identified different spheres in which patients can experience movement between existential uncertainty and certainty, implying opportunities for personal learning and growth. It is therefore important for healthcare professionals to be alert to situations where patients diagnosed with advanced disease can familiarise themselves with their changed bodies resulting from the disease, the day-to-day of everyday situations, companionship with others, the natural environment and different healthcare situations. Acknowledging and affirming how the individual in such a variety of spheres lives an uncertain future and confronts his or her personal impending death can pave the way for personal growth. Further research with explicit focus on existential uncertainty and certainty is merited. These results highlight the necessity of including existential dimensions of palliative care in clinical guidelines and policies for palliative care, in line with palliative care philosophy and theory.

## Competing interests

The authors declare that they have no competing interests.

## Authors’ contributions

JÖ, FF designed the project and secured project funding. JÖ and FF conducted all the interviews. CW made an initial processing of the data. MK analysed and interpreted the data. MK made a first draft of the manuscript. All authors contributed to data analysis and helped in revising and making substantial contributions to the manuscript, and also read and approved the final manuscript.

## Pre-publication history

The pre-publication history for this paper can be accessed here:

http://www.biomedcentral.com/1472-684X/13/28/prepub
